# Research on the Cognitive Diagnosis of Chinese Listening Comprehension Ability Based on the G-DINA Model

**DOI:** 10.3389/fpsyg.2021.714568

**Published:** 2021-09-07

**Authors:** Li Li, Yi An, Jie Ren, Xiaoman Wei

**Affiliations:** ^1^Collaborative Innovation Center of Assessment for Basic Education Quality, Beijing Normal University, Beijing, China; ^2^Institute of Language Testing and Talent Evaluation, Faculty of Linguistic Sciences, Beijing Language and Culture University, Beijing, China

**Keywords:** fine-grained evaluation, Chinese as a second language, G-DINA model, cognitive diagnosis theory, Chinese listening comprehension ability

## Abstract

As a new generation of measurement theory, cognitive diagnosis theory shows significant potential and advantages in educational evaluation in that it combines a cognitive process and a measurement method. The application of the theory not only reveals the potential characteristics of learners in cognitive processing, but also provides targeted remedies and strategic guidance for individuals. Given the difficulties of traditional assessment models in providing an insightful and fine-grained account for individualized and procedural learning, providing personalized learning strategies for learners of Chinese as a second language has been a new goal of teaching and measurement in Chinese listening. This study constructs a cognitive diagnosis model of Chinese listening comprehension for Chinese-as-a-second-language learners through theoretical exploration, model hypotheses, repeated verification, and model modification. The results show that the Q-matrix (Q_3_) constructed by the experts within modification has the highest fitting degree with the empirical data. The parameter recovery rate, the accuracy of the tested attribute or mode, and the relative fitting index obtained from the simulation study are consistent with the information extracted from the empirical data. The diagnostic reliability and effectiveness of generalized deterministic inputs, noise “and” gate (G-DINA) are higher than those of DINA, deterministic inputs, noisy “or” gate (DINO), and reduced reparametrized unified model (RRUM). In the estimation of the item and subject parameters, the G-DINA model shows good convergence, and the average classification accuracy rate based on attribute level is 0.861.

## Introduction

The famous British linguist Carl Weaver once said, “listening is the core of communication” (Goldhaber and Weaver, 1968; Zhou, 2000), and listening comprehension is the foundation of language learning. In 1978, Rivers et al. conducted a survey on the listening, speaking, reading, and writing activities of native English-speaking adults and found that listening accounted for up to 45% of human daily communication activities (Rivers and Temperley, 1978; Wang, 2011). Regarding Chinese-as-a-second-language (referred to as CSL) learners, Chinese listening ability plays an irreplaceable role in language communication. However, contrary to actual needs, academic research on listening comprehension ability, especially Chinese listening comprehension ability, is relatively scarce. In most teaching, listening class is always put in second place. There is a certain degree of blindness in the teaching, learning, measurement, and evaluation of Chinese listening ability, as well as in feedback and remedy.

Traditional psychological and educational measurement theory is not fine-grained enough to evaluate cognitive skills. We do not know the internal psychological processing, skills, strategies, and cognitive structures behind the scores. With the development of scientific research and social life, people are no longer satisfied with the practice of testing at the overall ability level. In 2002, the United States Congress passed the “No Child Left Behind Act” proposed by then President George W. Bush, which strengthened the role of tests in educational evaluation and the link between outcome evaluation and teaching. The act said that test without diagnosis and diagnosis without remedial teaching were both irresponsible. Educational evaluation should better reflect the learning of students, provide feedback for teaching, and integrate accountability testing, formative assessment, and professional support (Bennett and Gitomer, 2009). At present, there are few research studies and practices about this in Chinese measurement and evaluation. Therefore, in order to promote student development and ensure the quality of educational examinations, scientific and fine-grained evaluation and diagnosis must be conducted as soon as possible.

As a new generation of measurement theory, cognitive diagnosis shows a great potential and advantages. Among many cognitive diagnosis models, the generalized deterministic inputs, noisy “and” gate (G-DINA) model is general and saturated, which can subsume both compensatory and non-compensatory models (Sorrel et al., 2017). In a G-DINA model, each cognitive attribute of the item has a different contribution ratio to the probability of answering the item correctly, and students who only master some cognitive attributes also have a certain probability to get the correct answer. Besides, the parameter estimation of a G-DINA model includes not only all parameters with one single attribute, but also the interaction parameters among multiple attributes. In this way, it is in accordance with the comprehensiveness and diversity of the language test. Therefore, this study uses the G-DINA model to construct a structural model of listening comprehension in CSL examination. Empirical exploration can provide guidance and reference for language tests with Chinese as a second language, such as the Chinese Proficiency Test (HSK), National Vocational Chinese Proficiency Test (ZHC), and Chinese Proficiency Test for Minorities in China (MHK). For individuals, cognitive diagnostic information can help students better understand the knowledge they have mastered and tailor their learning plans. After summarizing the general patterns and problems in mastering cognitive attributes for examinees with different abilities, teachers can also propose personalized remedies according to different situations of examinees and then provide targeted guidance for subsequent Chinese teaching. For the country, the diagnosis report of students can reflect the current educational situation and provide a basis for educational planning and decision-making.

## Theoretical Foundations

### Derivation and Verification of Attributes and Q-Matrix

Attribute is a very important concept in cognitive diagnostic assessment (Leighton and Gierl, 2007; Von Davier, 2007). It is generally believed that attributes, represented by vector α, are cognitive skills, specific knowledge, or problem-solving strategies required from students to finish a certain test task (if there is no special explanation, attributes and skills are synonymous in this study). “Q-matrix” is an association matrix describing the relationship between test items and test attributes, and it is a bridge between the answers of students and their attribute mastery patterns.

In past research, attributes and the Q-matrix were mostly defined by domain experts, but this method is prone to inconsistent expert opinions, difficulties in establishing selection criteria encountered by experts, and inconsistent results between expert calibration and actual examinations. The mis-defined Q-matrix will have a profound impact on the estimation of model parameters and accurate classification of students (de la Torre and Chiu, 2016). With the need to construct a large-scale test item bank, it is time-consuming and laborious to calibrate the attributes of the items in the database individually through expert discussion. Some studies show that, in some cases, the Q-matrix estimated and calibrated by computers performs better than the Q-matrix defined by experts, and the practice of constructing the Q-matrix for cognitive diagnosis only through expert definition needs further discussion and improvement (Torre, 2008; Liu et al., 2011; Liu and Tu, 2015; Li, 2016).

**Figure 1:**
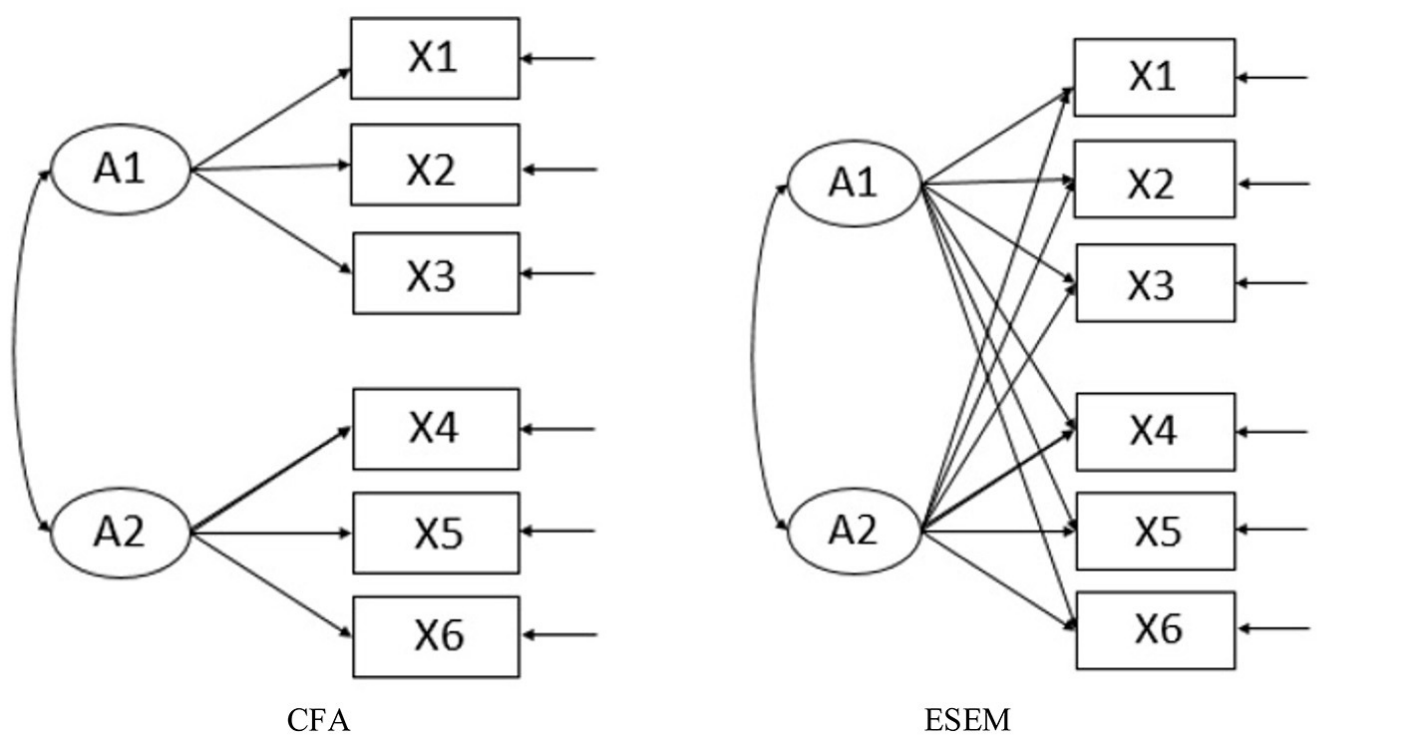
Model setting based on confirmatory factor analysis (CFA) and exploratory structural equation modeling (ESEM).

Recently, some scholars have tried to use confirmatory factor analysis (CFA) methods to verify the rationality of the Q-matrix, but the strong hypothesis of CFA does not apply to the conditions in most practical tests. This will bring a series of problems to model fitting and estimation. The exploratory structural equation modeling (ESEM) method uses part of a measurement model similar to exploratory factor analysis (EFA) and integrates the advantages of CFA. It allows free estimation of the load of each item on each special factor (Mai and Wen, 2013). Figure 1 shows the model settings based on CFA and ESEM. In this study, the ESEM method is used to construct the Q-matrix.

### Generation and Parameter Estimation of the G-DINA Model

In the cognitive diagnostic assessment process, the choice of model is very important. More than 60 types of cognitive diagnosis models have been developed thus far (Chen and Zhang, 2010; Zhang and Sha, 2013), but most of the existing models divide students into two potential categories: students who have mastered all attributes and those who have not mastered all the attributes. Only one of the attributes contained in an item is not mastered, which is equivalent to the category in which all the attributes are not mastered, such as in the deterministic inputs, noisy “and” gate (DINA) model (Junker and Sijtsma, 2001). This scoring method does not consider the interactive relationships among the attributes, which may cause the loss of diagnostic information. In language tests, due to the comprehensiveness and diversity of language, the measured skills and attributes are often multidimensional and multilevel. The relationship among the attributes is very close. Different language skills are related and can compensate for each other. Therefore, cognitive diagnosis requires a high-quality diagnosis model (de la Torre and Douglas, 2004; Chen, 2015; Gao et al., 2018). The G-DINA model is a generalized DINA model (de la Torre, 2011; Chen et al., 2013). Similar to the DINA model, the G-DINA model also requires a J × K Q-matrix. However, its hypothesis is more relaxed, so it can make a more flexible estimation of cognitive attributes. The mathematical expression formula is as follows:

(1) $$\text{p}(\alpha_{\text{lj}}^{*})=\delta_{\text{j}0}+\sum_{\text{k}=1}^{{\text{k}}_{\text{j}}^{*}}\delta_{\text{jk}}\alpha_{\text{lk}}+\sum_{\text{k}\prime =\text{k}+1}^{{\text{k}}_{\text{j}}^{\text{*}}}\sum_{\text{k}=1}^{{\text{k}}_{\text{j}}^{*}-1}\delta_{\text{jkk}\prime }\alpha_{\text{lk}}\alpha_{\text{lk}\prime }...+\delta_{\text{j}12...{\text{k}}_{\text{j}}^{*}}\prod_{\text{k}=1}^{{\text{k}}_{\text{j}}^{*}}\alpha_{\text{lk}}$$

The G-DINA model divides students into $2^{K_{j}^{*}}$ potential categories, where $P\left(\alpha_{lj}^{*}\right)$ represents the probability of correctly answering an item *j*. δ_*j*0_ is the intercept of item *j*, representing the benchmark probability of a correct answer (the probability of guessing the correct answer). δ_*jk*_ is the main effect of attribute α_*k*_, representing the influence of mastering a single cognitive attribute on the probability of a correct answer. $\delta_{jkk\prime }$ is the first-order interaction effect of attributes α_*k*_ and $\alpha_{k\prime }$, representing the influence of the relationship between the attributes on the probability of a correct answer. $\delta_{j12\ldots K_{j}^{*}}$ is the common interaction effect of attribute $\alpha_{1},\ldots ,\alpha_{K_{j}^{*}},$ representing the common influence of mastering all cognitive attributes on the probability of correct answers and exceeding the additional influence of the interaction between the main attributes and lower-order attributes.

## Prestudy

### Research Purpose

The determination of cognitive attributes, the validity of the Q-matrix, and the choice of diagnostic model are very important in cognitive diagnosis. This section mainly discusses the construction and verification of cognitive attributes and the Q-matrix of the CSL listening test. Simulation experimental research is performed to screen the best cognitive diagnosis model for the empirical analysis of cognitive diagnosis.

### Materials and Methods

The research takes a domestic Chinese proficiency test as the research basis. This test is authoritative in China. It includes four parts: listening, reading, speaking, and writing. The reliability and validity of the test have been tested. The research selects all the listening comprehension questions and the data of 35,031 valid candidates. The listening comprehension consists of three parts, with a total of 40 items, and the structure of the test is shown in Table 1. The listening attributes involved in the test questions are relatively proportionate.

**Table 1:** Listening test structure.

**Item**	**Content**		
Construction basis	Chinese proficiency listening syllabus and listening test theory		
Test structure	Part 1	Part 2	
	Single-round conversation	Multiple rounds of dialogue	Discourse
Item number	1–15	16–24	25–40
Item type	Multiple choices		
Completion time	30 min		

The “GDINA” package in the R software is used to estimate the parameters of the candidates and items.

### Results

#### Construction and Verification of Cognitive Attributes

From a linguistic perspective, based on the process model and component model of listening comprehension, this study divides the theoretical framework of listening attributes into two levels: language skills and cognitive strategies (Lado, 1961). From the perspective of cognitive psychology, the theoretical framework can be divided into three levels: surface coding, basic textual representation, and context model. Seven cognitive attributes for CSL listening ability have been preliminarily determined: ① understand the meaning of keywords, ② understand the meaning of sentences, ③ recognize context, ④ locate facts and details, ⑤ summarize the main idea, ⑥ inductive inference, ⑦ and short-term memory (see Table 2). Language skills and cognitive strategies are parallel to each other, and participants can use cognitive attributes at both levels at the same time.

**Table 2:** Definition of Chinese-as-a-second language (CSL) listening cognitive attributes.

**Language framework**	**Cognitive category**	**Attributes**	**Name**	**Definition**
Language skills	S	A1	Understand the meaning of keywords	Identify less commonly used vocabulary and expressions, common spoken phrases and idioms, etc. Understand the meaning of what you are listening to and can quickly perform synonymous conversion to match the correct option.
	T	A2	Understand the meaning of sentences	Use conjunctions, grammatical relationships or sentence structure to provide information and clarify the logical relationships between sentences, such as transition, progression, cause and effect, and conditions.
	SM	A3	Recognize context	Experience the context and infer the emotional attitudes and character relationships outside the speaker's words in the context.
Strategic skills	S	A4	Locate facts and details	Can selectively grasp the facts in the listening materials through the information given in the topic, background sounds, etc.; understand the expression of time, place and relationship; and capture detailed information at the same time.
	T	A5	Summarize the main idea	Summarize the main points and points of the dialogue or chapter.
	SM	A6	Inductive inference	Through background knowledge and previous textual information, it can be inferred that the speaker expresses the implicit hypothesis, the implicit meaning of the sentence, the speaker's purpose and intention, and conclusions.
	SM	A7	Short-term memory	Acquire information through short-term memory to complete the corresponding task.

*S, surface coding; T, basic textual representation; and SM, context model*.

**Figure 2:**
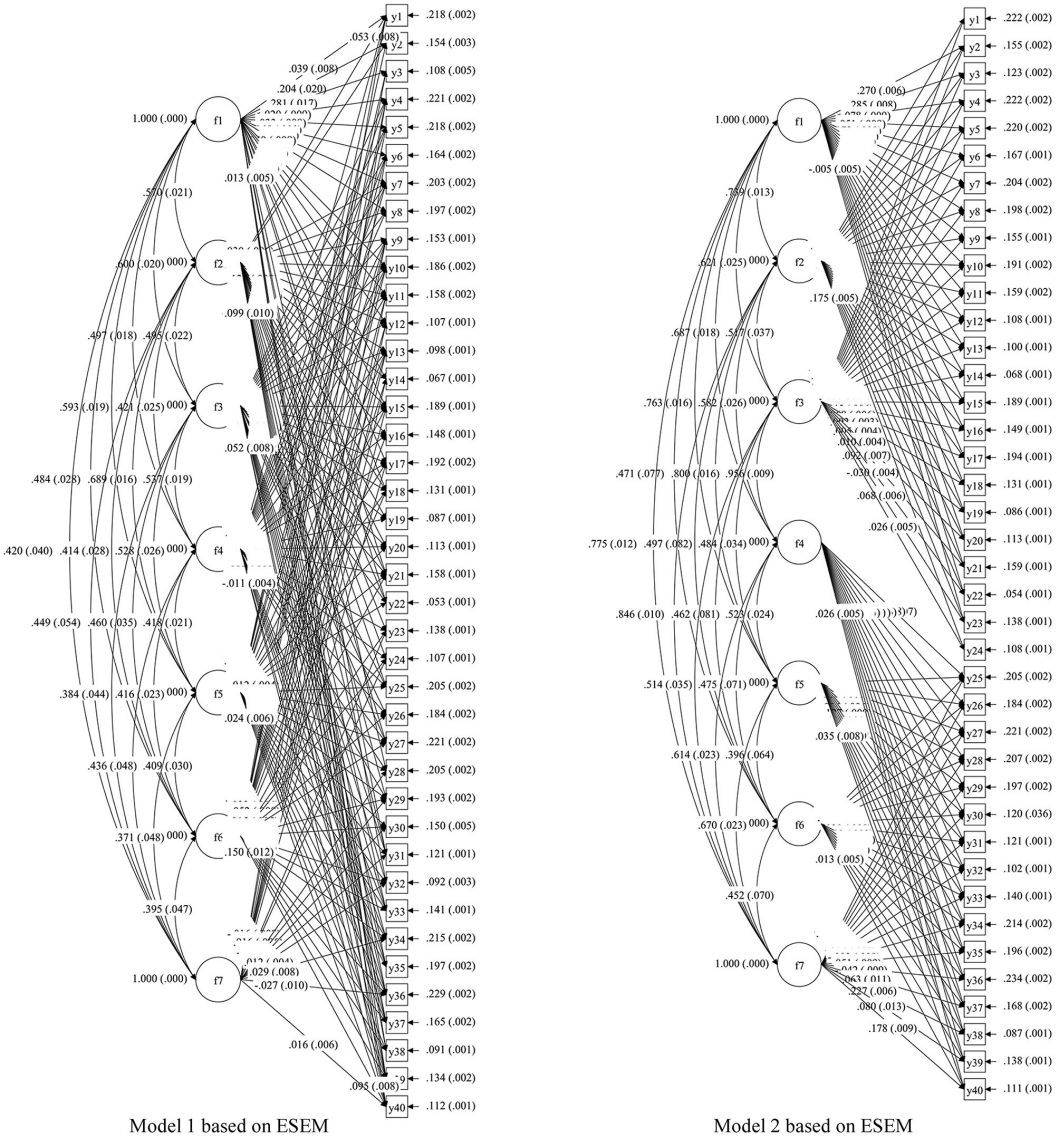
Structural diagram of two hypothetical listening cognitive models.

We recruited three experts in this field to demonstrate the cognitive attributes of Chinese listening. Two views have been proposed: one was that the cognitive attributes of language skills and strategic skills should be used in single-round dialogue, multi-round dialogue, and listening discourse questions. The other was that in the first part of listening comprehension (single-round dialogue and multi-round dialogue), the cognitive attributes at the language skill level should mainly be examined, and in the second part (listening discourse), the cognitive attributes at the strategic skill level should mainly be examined. In order to determine the relationship among the cognitive attributes of listening comprehension, the exploratory structural equation modeling (ESEM) method was used to compare the fitting degree in the two hypothetical models and data, so as to find the most reasonable cognitive model for this CSL listening test. The research uses the MPLUS8 software to define F1, F2, F3, F4, F5, F6, and F7 as the seven potential factors (listening attributes) of the CSL listening test, and Y1–Y40 are the sub-item codes of the listening test. The modeling information of the two views is shown as Models 1 and 2 in Figure 2, and the fitting degree of the data and model is shown in Table 3.

**Table 3:** Model fit.

	**χ^2^**	**df**	**χ^2^/df**	**TLI**	**CFI**	**AIC**	**BIC**	**SRMR**	**RMSEA**
Model 1	1593.847	521	3.059	0.992	0.995	1445116.257	1447985.549	0.006	0.008
Model 2	3080.215	641	4.805	0.986	0.988	1446362.625	1448216.238	0.008	0.010

For the Chinese-as-a-second-language listening test, the standardized root mean residual (SRMR) is <0.08, the root mean square error of approximation (RMSEA) is <0.05, and the comparative fit index (CFI) and Tucker-Lewis index (TLI) are >0.9, which means that Models 1 and 2 fit the data well. However, the Akaike information criterion (AIC) and Bayesian information criterion (BIC) of Model 1 are significantly smaller than those of Model 2 (the difference is more than 10), so Model 1 is more suitable for the sample of this study. Therefore, we believe that the seven attributes determined by the experts are all investigated in all item types of the CSL listening test, and the next step of Q-matrix construction will be conducted accordingly.

#### Construction and Testing of Q-Matrix

To construct the Q-matrix, the combination of qualitative and quantitative analyses is used for repeated verification. It is assumed that there is no fixed hierarchical relationship between the seven cognitive attributes tested in the CSL listening test (Liu and Tu, 2015). Five different versions of the Q-matrix were constructed based on this: the Q-matrix marked by the researcher according to the hypothetical model (Q_1_); the Q-matrix marked by 10 domain experts according to the hypothetical model (Q_2_); the Q-matrix modified according to the discriminant index ς2 based on Q_2_ (Q_3_) (de la Torre and Chiu, 2016); the Q-matrix marked according to the ESEM factor load (Q_4_), and the Q-matrix modified according to the discriminant index ς2 based on Q_4_ (Q_5_). More details about the construction method of different versions of the Q-matrix are shown below. According to the above five hypotheses, the model-data fitting test was conducted, and the optimal model that fits the data was selected.

Q_1_: The researcher determined Q_1_ through item analysis and model verification of Chinese listening comprehension. See Q_1_ in Appendix Table 1.

Q_2_: Ten experts in the field of teaching Chinese as a foreign language, linguistics and applied linguistics, educational measurement, and Chinese test proposition were assigned to mark the Q-matrix. The specific operation process was as follows: play the listening test file for each expert, provide a list of cognitive attributes prepared in advance, ask them to check the attributes that they think may be involved in each item, and sort the attributes according to the importance of the tested attributes. The cognitive attribute judgment of all items was completed independently, and discussion and communication were not allowed. The list included the seven cognitive attributes discussed before, and the opinions of the 10 experts and scholars were counted: if five or more experts (50% or more) mark the same attribute in the same item, the item would be considered to measure the attribute (marked as 1), while if four or less of the experts do so, the item would be considered not to measure the attribute (marked as 0). Finally, the experts constructed form Q_2_. See Q_2_ in Appendix Table 2.

Q_3_: Although the construction of a Q-matrix is usually performed by domain experts, to a large extent, it is still subjective. If it is not controlled, the mis-specified Q-matrix may cause deviation in the results of cognitive diagnosis. In order to solve this problem, we used the discrimination index ς2 to verify the validity of the Q-matrix by identifying and replacing the mis-specified items. With greater applicability and universality, ς2 generalizes the discrimination index ϕ proposed by Torre (2008) for DINA models (de la Torre and Chiu, 2016). The proportion of variance accounted for (PVAF) by a particular q-vector relative to the maximum ς2 was used as a criterion to select the q-vector for each item, and the cut-off point of the PVAF was set at 0.95 according to de la Torre and Chiu (2016). De la Torre and Chiu tested the feasibility of this method in a simulation study. The results showed that this method could accurately identify and correct the mis-specified Q-matrix items without changing the correct items. In this study, according to the initial Q-matrix constructed by the experts (Q_2_), a modified Q-matrix is obtained. See Q_3_ in Appendix Table 3.

This method will not replace the current method of constructing and verifying a Q-matrix. Instead, it aims to provide supplementary information to improve model-data fitting, so as to improve the effectiveness of inference in cognitive diagnostic assessment. The modified Q-matrix can be supplemental information to experts, and judgments based on domain knowledge are always needed. It should be noted that in many applications, the Q-matrix constructed based on the validation method may be different from the Q-matrix constructed based on experts, sometimes significantly different. Therefore, it is of great value to study how to deal with these differences.

Q_4_: Due to the diversity, comprehensiveness, abstraction, and being indistinguishable of language tests, an item may test several different cognitive attributes at the same time. Therefore, the construction of a Q-matrix by confirmatory factor analysis may be inconsistent with the actual situation. Because the assumptions of CFA model are often too idealized, the model assumes that each variable measures only one factor, in which the cross loading is limited to 0 and the residual correlation of index variables is 0. These strong assumptions and limitations inconsistent with the real test will bring a series of problems to the fitting and estimation of the model, resulting in a large number of model modifications, distortion of the factor structure, overestimation of the correlation between factors, etc. Therefore, this study intends to perform exploratory structural equation modeling (ESEM) with both the exploratory factor analysis (EFA) and confirmatory factor analysis (CFA) functions to construct Q_4_. Because the ESEM method assumes continuous latent variables and the cognitive diagnosis modeling method assumes dichotomy attributes, the discrete factor loading (DFL) method is used to discretize the factor loading matrix estimated by ESEM to obtain a binary Q-matrix. We chose the row average factor loading as the threshold for discretizing continuous variables into a binary Q-matrix. Wang et al. (2018) showed that the DFL method could mine information from data and provide a high-quality correct recovery rate (CRR) based temporary Q-matrix (Wang et al., 2018). Mplus 8.0 was selected for parameter estimation and data analysis. In addition, statistical optimality does not mean that the model is the most appropriate. Therefore, the Q-matrix constructed with the ESEM method should be used carefully, and the content of the item should be analyzed further. See Q_4_ in Appendix Table 4.

Q_5_: Q_5_ adopted the same method of constructing Q-matrix as Q_3_, but the initial Q-matrix of Q_5_ is Q_4_. See Q_5_ in Appendix Table 5.

##### Relative Fitting Test

At the test level, the Akaike information criterion and Bayesian information criterion were used to check the fitting index of the four different cognitive diagnosis models and data with different Q-matrices. The smaller AIC and BIC are, the better the model fits the data (Chen et al., 2013). Table 4 shows that the modified Q_3_ fits each cognitive diagnosis model well, and the fitting index with the G-DINA model is the best. Therefore, we will analyze and discuss the statistical results of cognitive diagnosis based on the Q_3_ model in the following steps.

**Table 4:** Relative fit evaluation index at the test level.

	**Fitting Index**	**Q-matrix Determined by the Researcher (Q_**1**_)**	**Expert Q-Matrix (Q_**2**_)**	**Revised Expert Q-Matrix (Q_**3**_)**	**ESEM Builds Q-Matrix (Q_**4**_)**	**Revised ESEM-Q-Matrix(Q_**5**_)**
G-DINA	−2LL	1453717.84	1442296.22	1439148.58	1441999.6	1441693.46
	AIC	1454315.84	1443086.22	1439854.57	1442825.60	1442491.47
	BIC	1456846.58	1446429.50	1442842.36	1446321.22	1445868.60
DINA	−2LL	1473158.8	1473215.88	1475330.42	1465608.86	1465543.14
	AIC	1473572.81	1473629.88	1475744.42	1466022.85	1465957.14
	BIC	1475324.85	1475381.92	1477496.47	1467774.90	1467709.18
DINO	−2LL	1475386.24	1475414.5	1474199.36	1466650.08	1466113.5
	AIC	1475800.24	1475828.50	1474613.35	1467064.07	1466527.50
	BIC	1477552.28	1477580.55	1476365.40	1468816.12	1468279.55
RRUM	−2LL	1456796.96	1448627.6	1446451.8	1444293.28	1456796.96
	AIC	1457288.95	1449163.60	1446977.80	1444829.28	1457288.95
	BIC	1459371.10	1451431.95	1449203.83	1447097.63	1459371.10

##### Absolute Fitting Test

The root mean square error of approximation was selected as the absolute fitting index based on the test level. In general, RMSEA <0.06 and SRMR <0.08 indicate that the model and data fit well. The revised expert Q-matrix (Q_3_) (RMSEA = 0.01, SRMR = 0.013) fits the sample well.

#### Choice of Cognitive Diagnosis Model

In order to investigate the applicability of the generalized deterministic inputs, noisy “and” gate model in language testing, we conducted an empirical study and a simulation study at the same time.

##### Empirical Study

The performance of different cognitive diagnosis models in empirical data are compared by fitting statistics at the whole test and item levels. The estimation of fitting statistic based on likelihood ratio test shows that the G-DINA model as the zero model is statistically significant compared with DINA, deterministic inputs, noisy “or” gate (DINO), and reduced reparametrized unified model (RRUM), *p* < 0.001 (see Appendix Table 6 for details). The G-DINA model cannot be replaced by the three other models. It should be noted that this model-data fitting statistics is very sensitive to sample size. When the sample size increases, null hypothesis will likely be overturned, and then poor model data fitting will be drawn. In addition, in the case of a large number of attribute mastery patterns, the model data fitting based on chi square has a high probability of making type I errors. Therefore, such fitting indicators should be used carefully in cognitive diagnostic assessment (Wang and Song, 2015). The fitting statistics based on item level includes: the log odds ratios for each item pair, the transformed correlation for each item pair, and the proportion correct statistics for each item.

*Log odds ratio for each item pair* It is the logarithm of an inter-item correlation index. $n_{k,k^{\prime }}$ is the person who gets k points on item j, and *k*′ points on topic *j*′, *k, k*′ = 0, 1. Item fitting can be evaluated by calculating the absolute difference between the logarithm occurrence ratio of item j and item *j*′ in the observation data and prediction data. The closer the mean value of $l_{jj^{\prime }}$ is to 0, the better the model fits the item j.

(2) $$l_{jj\prime =}\left|logOR-log\tilde{OR}\right|$$

(3) $$\begin{array}{l} OR=\frac{n_{11}n_{00}}{n_{10}n_{01}} \end{array}$$

*Transformed correlation for each item pair*$r_{jj\prime }$ is obtained by calculating the Pearson correlation coefficient between two items. *X*_*j*_ and ${\tilde{X}}_{j\;}$are the response vectors of item *j* in the observed data and expected data. The test of goodness-of-fit is performed by measuring the relevant differences between item pairs in the observed data and expected data. The formula is

(4) $$r_{jj\prime =}\left|Z\left[Corr\left(X_{j},X_{j\prime }\right)\right]-Z\left[Corr\left({\tilde{X}}_{j},{\tilde{X}}_{j\prime }\right)\right]\right|$$

where Corr() is Pearson correlation coefficient and Z() is Pearson's Fisher Z conversion value.

*Proportion correct statistics for each item*$l_{jj\prime }$ and $r_{jj\prime }$ are information based on an item pair. *p*_*j*_ represents the statistic based on the correct answer proportion for each item. It measures the difference between the correct answer proportion of a single item in the observation data and in the prediction data.

(5) $$p_{j}=|\sum_{i=1}^{N}\frac{X_{ij}}{N}-\sum_{i=1}^{N}\frac{{\tilde{X}}_{ij}}{\tilde{N}}|$$

It is found that statistics based on single item information is very poor in fitting test. Correspondingly, there is little difference in the fitting test performances of statistics based on item pair correlation and logarithm occurrence ratio (Chen et al., 2013). Table 5 shows the mean value, maximum value, and standard error of maximum value of the three item-fit statistics ($l_{jj\prime }$, $r_{jj\prime }$, and *p*_*j*_) under the different cognitive diagnosis models. With the significant level at 0.1 and Bonferroni correction tests, the means of $l_{jj\prime }$ and $r_{jj\prime }$ of the G-DINA model are 0.051 and 0.01, respectively, which are better than those of DINA, DINO and RRUM, and the means of the *p*_*j*_ of the four models are 0.001, 0.001, 0.001, and 0, respectively.

**Table 5:** Item-fit statistic based on four cognitive diagnosis models.

	**Item-fit statistic**	**l**	**r**	**p**
G-DINA	Mean	0.051	0.01	0.001
	Max	0.342	0.071	0.002
	SEmax	0.041	0.005	0.003
DINA	Mean	0.230	0.045	0.001
	Max	1.187	0.184	0.002
	SEmax	0.055	0.005	0.003
DINO	Mean	0.231	0.045	0.001
	Max	1.056	0.19	0.002
	SEmax	0.045	0.005	0.003
RRUM	Mean	0.089	0.018	0
	Max	0.208	0.136	0.002
	SEmax	0.029	0.005	0.002

##### Simulation Study

Using the G-DINA package of the R software, the seven attributes were considered independent with each other, and 2,000 samples were simulated. The number of items was fixed at 40, and the guessing and slipping parameters of each item were fixed (0.1, 0.1), (0.1, 0.3). According to the Q-matrix determined by the discussion of the experts, the score matrix of examinees was randomly generated by 10-time simulation under each condition, so were the attribute master pattern matrix and the item parameters. An expectation-maximization (EM) algorithm was used to estimate the item parameters of G-DINA, DINA, deterministic inputs, noisy “or” gate (DINO), and reduced reparametrized unified model (R-RUM).

The accuracy and applicability of the results of different cognitive models in simulation research were assessed by root mean square error (RMSE), mean absolute error (MAE), attribute accuracy, pattern accuracy, and relative fitting indicators.

*Root mean square error (RMSE) and mean absolute error (MAE)* The root mean square error index and absolute deviation index (mean absolute error) were compared with the estimated value and the true value of the simulated data. The calculated formulas are as follows:

(6) $$\begin{array}{l} RMSE=\frac{1}{N}\overset{N}{\underset{n=1}{\sum }}\sqrt{\frac{1}{R}\overset{R}{\underset{r=1}{\sum }}{\left({\hat{\varsigma }}_{r}-\varsigma_{r}\right)}^{2}} \end{array}$$

(7) $$\begin{array}{l} MAE=\frac{1}{N}\overset{N}{\underset{n=1}{\sum }}\frac{1}{R}\overset{R}{\underset{r=1}{\sum }}|{\hat{\varsigma }}_{r}-\varsigma_{r}| \end{array}$$

${\hat{\varsigma }}_{r}$ represents the parameter values estimated by the different cognitive diagnosis models. ς_*r*_ represents the true value of the parameter, R represents the number of items in the entire test, and N represents the number of repetitions. The smaller the RMSE (the typical cutoff point of using the RMSE to evaluate model fit is 0.05) and MAE are, the closer the model estimation of a parameter is to the true value, and the better the model and data fit. The results comparing the estimated results of different cognitive diagnosis models in the simulation study with the true values of the simulation parameters are as follows.

Table 6 shows that the root mean square error and mean absolute error of the G-DINA model are the smallest, and that the root mean square error and mean absolute error of the DINA model are the largest under each condition. Therefore, regarding the authenticity of the simulated parameters, the G-DINA model is the best choice for cognitive diagnosis in language tests.

**Table 6:** Parameters returned by different cognitive diagnosis models.

**Fixed parameter**	**Model**	**MAE**		**RMSE**	
		**Guessing**	**Slipping**	**Guessing**	**Slipping**
	G-DINA	0.1521	0.0205	0.0381	0.0286
Guessing = 0.1,	DINA	0.3599	0.1050	0.4319	0.1409
Slipping = 0.1	DINO	0.2196	0.1581	0.3185	0.1923
	RRUM	0.2191	0.0464	0.3202	0.0713
	G-DINA	0.0162	0.0758	0.3554	0.0533
Guessing = 0.3,	DINA	0.0214	0.0934	0.4102	0.1079
Slipping = 0.1	DINO	0.0208	0.1086	0.3815	0.1177
	RRUM	0.0195	0.0926	0.3673	0.0601

*Accuracy rate of attributes or patterns of subjects* The proportion of correctly classified attributes (PCAs) is the correct classification rate at the attribute level, and the proportion of correctly classified attribute vectors (PCVs) is the correct classification at the vector level rate (Ma et al., 2016). They are expressed by the following formulas:

(8) $$PCV=\frac{\sum_{r=1}^{Rep}\sum_{i=1}^{N}I[\alpha_{i}={\hat{\alpha }}_{i}]}{N\times Rep},\;and\;PCA=\frac{\sum_{r=1}^{Rep}\sum_{i=1}^{N}\sum_{k=1}^{K}I[\alpha_{ik}={\hat{\alpha }}_{ik}]}{N\times K\times Rep}$$

**Figure 3:**
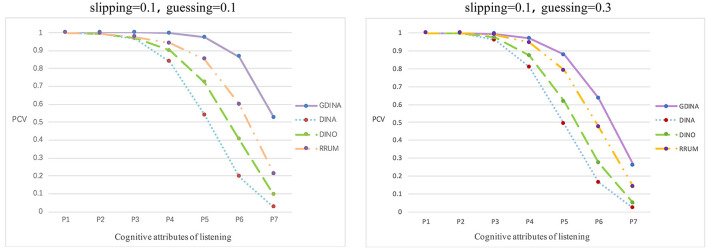
Average proportion of correctly classified attribute vectors.

**Figure 4:**
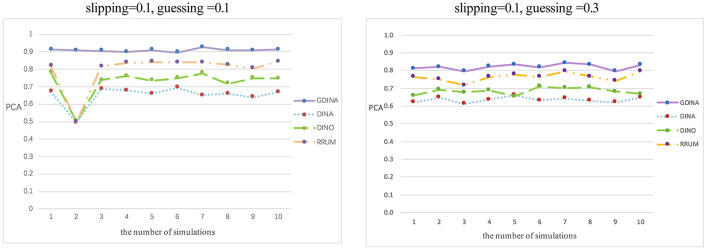
Classification accuracy rate of attribute level.

Rep represents the number of simulations, N is the number of participants, *i* refers to the *i*_*th*_ candidate, and K represents the number of simulated cognitive attributes. $I\left[\alpha_{i}={\hat{\alpha }}_{i}\right]$ and $I\left[\alpha_{ik}={\hat{\alpha }}_{ik}\right]$ indicate whether the estimated attribute vector is the same as the simulated true value and whether each attribute is the same as the simulated true value, respectively. The larger the PCVs and PCAs are, the more accurate the estimated parameters of the subjects will be. The classification accuracy rate of each model varies greatly. The G-DINA model has the highest classification accuracy rate in different parameter levels, reaching 90.87 and 80.14%, followed by R-RUM (79.61 and 76.32%) and DINO (72.59 and 68.33%). However, the DINA model has the lowest classification accuracy rate, and the proportion of correct classification attributes is only 65.06 and 63.46%. The average accuracy rate of the attribute vector and the accuracy rate at the attribute level are shown in Figures 3, 4.

Figure 3 shows the average classification accuracy rate of the attribute vector. P1 represents the proportion of at least one attribute in the true value of the simulated attribute mastering vector correctly recognized. P2 represents the proportion of at least two attributes in the true value of the simulated attribute control vector correctly recognized, etc. P7 represents the proportion of all elements in the attribute master vector being correctly identified. Overall, as P increases, the average accuracy rate of the attributes of the four cognitive diagnosis models shows a downward trend, which means that for any model, the higher the number of correctly identified attribute vectors is, the lower the accuracy rate will be. However, in general, the average accuracy rate of the attributes of the G-DINA model is higher than that of the three other models. Figure 4 shows the classification accuracy rate at the attribute level in each simulation. Based on Figure 4, the classification accuracy rate is the most stable in the G-DINA model, and the rate is ~0.9, higher than that in the three other models, while the rate is lowest and least stable in the DINA model.

*Relative fitting index* Relative fitting indexes of the different cognitive diagnosis models are shown in Table 7. The results demonstrate that the G-DINA model fits the data best, the R-RUM model is second, and with a relatively large AIC and BIC, the DINA model does not fit well. Because the DINA model is non-compensated, it can be preliminarily inferred that the attributes are compensated in the language test. Because the G-DINA model assumes that the attributes can be compensated for each other, it has less bias in data analysis in language tests.

**Table 7:** Relative fitting indexes of the different cognitive diagnosis models.

**Diagnostic model**	**Compensation between attributes**	**Guessing = 0.1, Slipping = 0.1**		**Guessing = 0.3, Slipping = 0.1**	
		**AIC**	**BIC**	**AIC**	**BIC**
G-DINA	Compensation	86870.06	89082.42	92587.17	94799.52
DINA	Non-compensation	94763.57	95922.96	95263.57	96422.96
DINO	Compensation	93347.34	94506.73	94807.67	95967.05
R-RUM	Compensation	89852	91353.04	93381.82	94882.86

## Empirical Research

### Research Purpose

According to the results of the study, the best performing model, G-DINA is selected for the empirical analysis of cognitive diagnosis. We can obtain the statistics of the test, items, and examinees, such as the probability of mastering attributes based on the group and individual levels, the estimated item parameters, and attribute classification accuracy.

### Materials and Methods

The performance data of 35,031 examinees on the seven cognitive attributes of Chinese listening are included. The G-DINA model in the R software was used for statistical analysis.

### Results

#### Reliability and Validity of the Test

Reliability and validity are the key indicators to measure the quality of a measurement tool. The cognitive diagnostic test reporting individual scores is set not to rank them but to provide the attribute classification results with criterion-referenced interpretations. Some researchers believe that the reliability of diagnostic scores is difficult to guarantee, while others believe that the diagnostic information provided by cognitive diagnostic assessment is irreplaceable in improving teaching and learning. Reliability and validity research based on cognitive diagnosis theory is still a relatively new research field. Reliability is often not reported in diagnostic scores reports. At present, two evaluation indicators evaluating the reliability and validity of diagnosis results are the classification consistency index and classification accuracy index.

The classification accuracy index of attributes or patterns can help to estimate the accuracy of simulated attributes or patterns, because the accuracy rate is usually unknown in real tests. In this case, using the classification accuracy index to evaluate the accuracy of a real test has an important application value for reliability and validity in educational evaluation (Wang et al., 2015). Based on the empirical analysis results of the G-DINA model, this study also provides classification accuracy based on three levels: the test level, the pattern level, and the attribute level. According to IRT theory, classification accuracy refers to the percentage of consistency between the observed and expected proportions of candidates in each category. The classification accuracy index of an attribute or a pattern can be obtained in three steps: (1) the MAP method with the G-DINA package can be used to estimate the parameters, (2) the expected classification probability of the attribute for each student is calculated according to the likelihood function of students, and (3) the classification accuracy index at the attribute or pattern level is estimated by calculating the percentage of consistency between the observed classification pattern and the expected classification pattern (Wang et al., 2015).

① Classification accuracy based on the test level
  Test level accuracy = .6263
② Classification accuracy based on the pattern level
③ Classification accuracy based on attribute level

The classification accuracy based on the pattern level is shown in Appendix Table 7 clearly. It is no longer listed in the text because of limited space. A summary of the classification accuracy of the seven attributes using kappa (Cohen, 1960) is shown in Table 8. It is generally believed that a value higher than 0.6 indicates basic consistency, and that a value higher than 0.8 is considered to be a near perfect cognitive diagnosis analysis result. The accuracy of the seven attributes ranges from 0.8 to 0.95, which should be considered almost perfectly consistent. According to the above criteria, the cognitive diagnosis analysis using the G-DINA model in this study is high in attribute classification, indicating that the diagnosis is highly reliable and valid.

**Table 8:** Classification accuracy based on the attribute level.

	**A1**	**A2**	**A3**	**A4**	**A5**	**A6**	**A7**
Probability	0.8045	0.8362	0.9255	0.9379	0.8677	0.8070	0.8494

The low classification accuracy of some attribute patterns may be resulted from the low distribution probability of these attribute patterns. In all the samples based on empirical data, only a very small number of examinees are classified into these attribute patterns. Too small sample size may lead to biases in the identification of cognitive diagnostic attribute mastery patterns. There are seven cognitive attributes in Chinese listening comprehension. Based on the empirical data, 35,031 examinees have produced 77 different kinds of attribute mastery patterns. The attribute mastery pattern “0011100” accounts for the largest proportion (23.26%), followed by “1111111” (22.25%), “0100011” (13.79%), and “0000000” (9.29%). These four attribute patterns are also high in classification accuracy. This shows that the language skills are interrelated, especially between A3, A4, and A5, and A2, A6, and A7. In this way, the information obtained from the G-DINA model can be used to give feedback to teachers and students.

#### Average Mastery Probability

**Figure 5:**
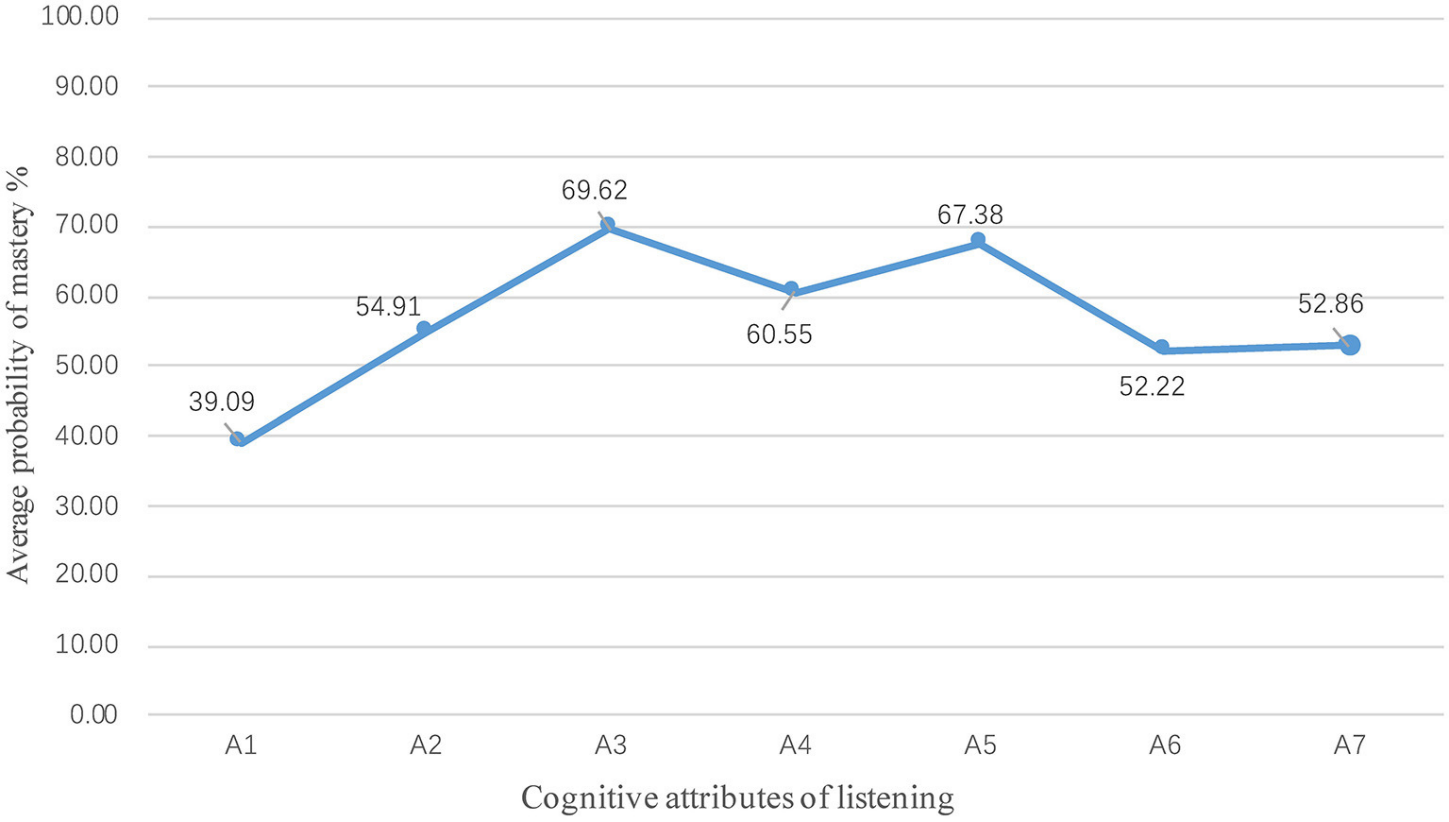
Average mastery probability at group level.

With the generalized deterministic input, noisy “and” gate model in R, statistical analysis of the performance of 35,031 students can be drawn in seven cognitive attributes of the Chinese-as-a-second-language listening test, then the mastery probability of each student in each attribute can be obtained, and so is the average group attribute mastery. Figure 5 shows the probability of mastering group attributes. Students had the highest grasp probability in identifying context (A3, 69.62%) and summarizing the main idea (A5, 67.38%).

**Figure 6:**
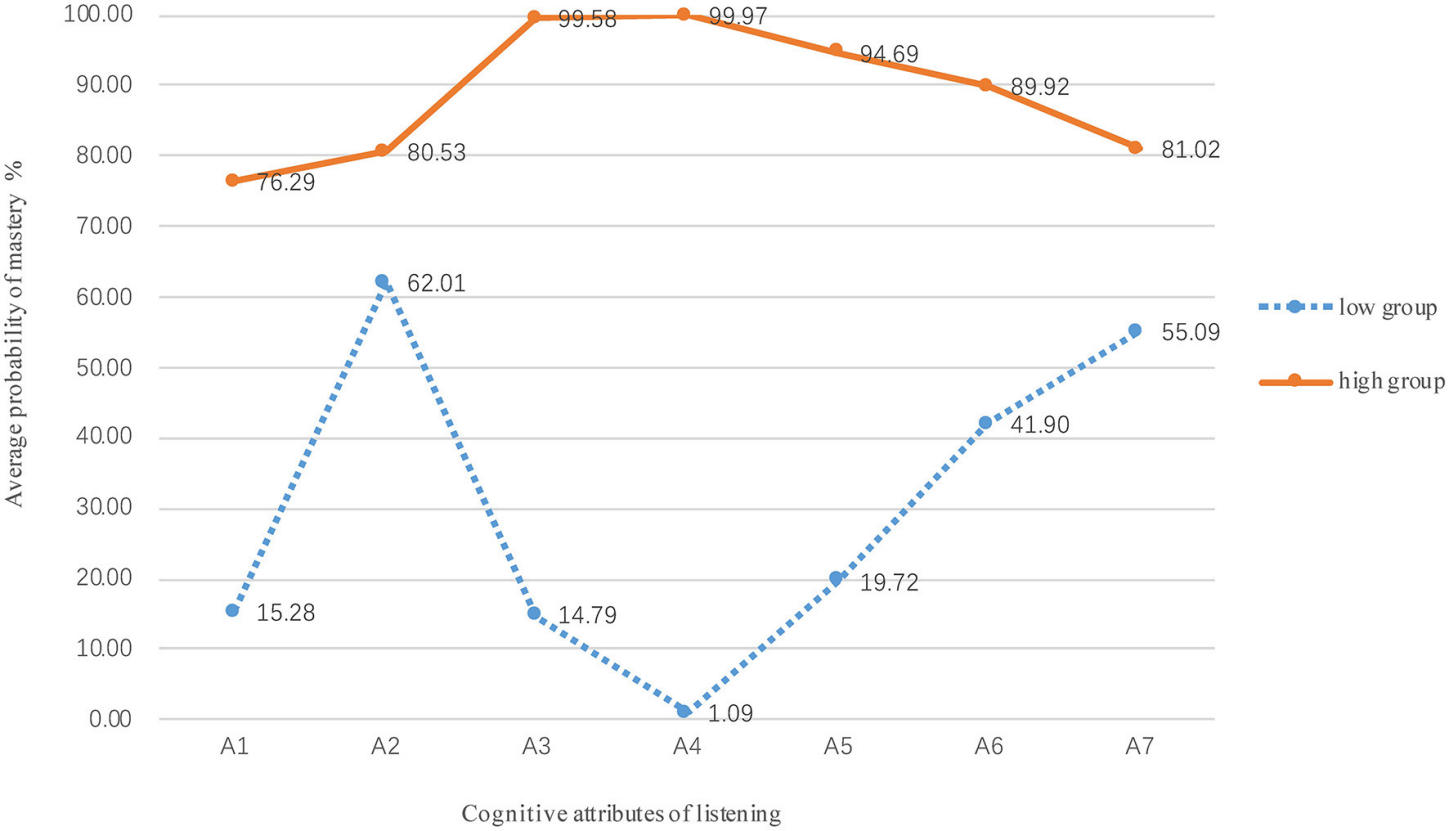
Average mastery probability between high and low groups.

In order to better understand the differences in the mastery of candidate groups with different ability, the 35,031 candidates were divided into groups with high and low levels to plot the average probabilities of mastery (Figure 6). Figure 6 shows that high-level candidates had the highest probability in mastering the recognition context (A3, 99.58%) and locating facts and details (A4, 99.97%). Almost all the high-level candidates have mastered attributes A3 and A4, but they did not perform well in the mastery probability of attributes of understanding words (A1, 76.29%) and sentence-level meaning (A2, 80.53%). However, the low-level candidates had higher probability in understanding sentence-level meaning (A2, 62.01%) and short-term memory (A7, 55.09%), but their probability of mastering facts and details (A4) was only 1.09%. The probability of mastering each attribute in the high-level group was relatively stable, while the probability of mastery in the low-level group fluctuated greatly, which indicates that some attributes may be easier to learn and master.

#### Estimated Item Parameters

Table 9 shows the item parameters based on the analysis of the generalized deterministic inputs, noisy “and” gate model. The data shown in the table represents the probability of successfully mastering the specific attribute of each item (that is, the probability of success when mastering one attribute required of the item, the probability of success when mastering two attributes required of the item,., and the probability of success when mastering all the required attributes of the item). A-P represents the attribute mastering pattern corresponding to items with different numbers of attributes. If the number of the required attributes for each item in the test is at most 4, then *K* = 4, so J^*^2^*k*^ = 40^*^16 item parameter values. For example, item 2 requires one attribute, items 4 and 7 require four attributes, and each of the remaining items requires two attributes. Although whether the item is mastered or not is represented by 1 and 0, respectively, the attributes required for each item are not the same. For example, items 6 and 17 also require two attributes, but the attributes required for the two questions are different (item 6 requires the two attributes of A3 and A6, and item 17 requires the two attributes of A4 and A7). The attributes required for each item are shown in the revised Q-matrix (Appendix Table 3).

**Table 9:** Estimation of the probability of answering the question correctly.

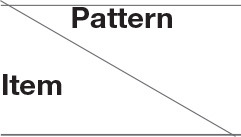	**Estimated probability of answering the question correctly**															
	**A**	**B**	**C**	**D**	**E**	**F**	**G**	**H**	**I**	**J**	**K**	**L**	**M**	**N**	**O**	**P**
1	0	1														
2	00	10	01	11												
3	000	100	010	001	110	101	011	111								
4	0000	1000	0100	0010	0001	1100	1010	1001	0110	0101	0011	1110	1011	1101	0111	1111
1	0.43	0.54	0.51	0.85												
2	0.28	0.84														
4	0.24	1.00	0.20	0.50	0.52	0.21	0.60	1.00	0.02	0.33	0.69	0.41	0.00	0.40	0.67	0.72
6	0.30	0.70	0.42	0.93												
7	0.23	1.00	0.56	0.00	0.62	0.33	1.00	1.00	0.77	1.00	0.77	0.69	0.31	0.65	0.89	0.68
......																
16	0.37	0.85	0.67	0.93												
17	0.12	0.33	0.18	0.47												
......																

For items that only require one attribute, such as item 2, P(0) and P(1) represent the guessing parameter (guessing) and 1-slipping parameter (slipping) in the deterministic inputs, noisy “and” gate model, respectively. According to the Q-matrix, P(α_3_ = 0, α_6_ = 0) of item 6 = 0.3 means that candidates who have not mastered the two attributes of A3 and A6 have a 30% probability of guessing the question correctly. P(α_3_ = 1, α_6_ = 0) = 0.7 means that candidates who have mastered attribute A3 but have not mastered attribute A6 have a 70% probability of answering this item correctly, P(α_3_ = 0, α_6_ = 1) = 0.42 means that candidates who have mastered attribute A6 but have not mastered attribute A3 only have a 42% probability of being able to answer question 6 correctly. P(α_3_ = 1, α_6_ = 1) = 0.93 represents that candidates who have mastered attributes A3 and A6 required for item 6 have a 93% probability of being able to answer the question correctly. This means that only one attribute (A3 or A6) is not very helpful in answering the question, but mastering two attributes at the same time can result in a 93% probability of answering the question correctly.

## Discussion

### Tetrachoric Correlation Among the Attributes

The tetrachoric correlation matrix among the seven attributes in the Chinese listening comprehension test can be obtained according to the generalized deterministic inputs, noisy “and” gate model. It can be seen from Appendix Table 8 that the positive correlations between A2 (understand the meaning of senses) and A7 (short-term memory), and A3 (recognize context) and A5 (summarize the main idea) are as high as 0.997 and 0.996, respectively. A1 (understand the meaning of keywords) has a medium-degree positive correlation with A2, A3, A5, and A7, which reflects the difficulty of distinguishing the listening skills. A4 (locate facts and details) has a low negative correlation with A2 and A7. A6 (inductive inference) has a moderate positive correlation with attribute A2, but its correlation with the other attributes is low or zero. Sometimes, it is negatively correlated with them. In general, the G-DINA model is sensitive to the relative independence and interrelation between the seven cognitive attributes of Chinese listening comprehension, which is consistent with the diversity and comprehensiveness of language skills and the complexity of related tests. At the same time, it also shows that the G-DINA model, which can subsume the compensatory and non-compensatory data perfectly well, has a high degree of consistency with the diversity of listening skills.

### Hierarchical Relationship Between Attributes

The hierarchical relationship among the attributes describes the topological order of the attributes in the field of testing, and on the other hand, it also describes the dependency relationship among the attributes, which is the basis of constructing a cognitive model. The attribute hierarchy model (AHM), as one of the cognitive diagnosis models, requires that attributes must have hierarchical relations (linear, convergent, branching, or unstructured), which is conducive to making the attributes and the cognitive process to be measured and inferred operable.

Although most scholars have realized the importance of the relationship between attributes in cognitive models, there is still a lack of effective cognitive models in the field of educational measurement, and some experts in cognition have said that this situation would not change in the near future, because we have known little about how a student truly knows the answer of a question. Thereupon, there is no corresponding cognitive model. Some studies have shown that the AHM assumes that all subjects are based on the same kind of processing strategy or problem-solving strategy. However, with the deepening of brain research, we have gradually found that this assumption is often inconsistent with the actual situation, because individual psychological processing characteristics vary, and processing strategies and problem-solving strategies are also different, thus the process of solving the same problem is not the same (Tu et al., 2012; Meng, 2013; Xie, 2014). Therefore, this study assumes that there is no fixed hierarchical relationship among the seven cognitive attributes tested in the listening test. Based on this assumption, different versions of the hypothesis model (Q-matrix) are constructed for this test.

### Model Selection

In order to investigate the applicability of the generalized deterministic inputs, noisy “and” gate model in the language listening testing, we conducted an empirical study and a simulation study at the same time. The fitting statistics based on the item-level in the empirical data shows that the G-DINA model fits best compared with the other cognitive diagnosis models. In the simulation study, the number of attributes was fixed at 7, Q_3_ was used as the initial matrix, and 2,000 samples were simulated. The number of items was fixed at 40, and the guessing and slipping parameters of each item were fixed at two levels (0.1, 0.1), (0.1, 0.3). The G-DINA model performs best at different item parameter levels, in that the parameter recovery, accuracy rate of the attributes or patterns of the subjects, and relative fitting index obtained from the simulation study are consistent with the information extracted from the empirical data. Therefore, the G-DINA model is demonstrated to be the best among the four cognitive diagnosis models and can be well-utilized in the analysis of Chinese listening comprehension test data.

### Limitations and Future Directions

Although the cognitive attributes and Q-matrix determined in this study have been verified by qualitative analysis and quantitative analysis, whether they can be extended to more general language proficiency tests need further in-depth research. In addition, the construction of the Q-matrix is an extremely difficult, laborious, and crucial problem. The wrong setting of the Q-matrix will bring undesirable diagnostic consequences. One limitation of this study is that a modified method of the PVAF method is adopted, and that the cut-off point is limited to 0.95 (Ma and Torre, 2020; Wang et al., 2020). The value is arbitrary to some extent, so it may be suboptimal under different conditions. For example, a ϕ as low as 0.75 has been shown to perform better under demanding conditions (e.g., low-quality items; Nájera et al., 2019). Nájera et al. (2021b) proposed a new Q-matrix verification method, Hull's method. The research shows that this method has greater flexibility and provides a comprehensive solution to Q-matrix specification (Nájera et al., 2021a,b). Therefore, it is necessary to explore the effectiveness of different Q-matrix modification methods in the cognitive diagnostic assessment of Chinese listening tests in the future research. Besides, how to evaluate the cognitive skills of students with the most appropriate granularity and how to explore the attribute-assisted calibration method based on data-driven calibration are the problems demanding deeper study and investigation by researchers. The ultimate goal of cognitive diagnosis is to give feedback information, including to put the results of educational measurement into practice directly for learners and teachers in teaching, learning and other practical activities, to understand the reasons that affect the probability of mastery of attributes by students, and to help principals, teachers, and students make better use of diagnostic information to improve teaching and learning efficiency. All of these issues entail further study by researchers in education and psychometrics.

## Conclusion

This research constructs a cognitive diagnosis model of Chinese listening comprehension ability for second language learners through theoretical exploration, model hypotheses, and repeated verification. In addition, we have found that the G-DINA model can more sensitively perceive the interrelationships between listening attributes, and the compensation and saturation of the model are consistent with the diversity and abstraction of listening skills in language ability, which demonstrate the unique advantages of the model in analyzing Chinese listening comprehension ability. The research results show that the constructed CSL listening comprehension cognitive diagnosis model is reasonable, and that the diagnosis results obtained with the G-DINA model have high reliability and validity. The results of this study are of guiding significance to Chinese listening teaching. Teachers can integrate the seven listening attributes into listening, teaching, and designing relevant exercises involving each attribute. Diagnostic information at the group level can be used by teachers and teaching administrators to adjust the syllabus and points in teaching CSL listening comprehension, and to formulate different intervention guidance for groups of students at different levels.

## Data Availability Statement

The raw data supporting the conclusions of this article will be made available upon reasonable request to the corresponding author.

## Author Contributions

LL prepared the first draft. YA, JR, and XW provided insightful comments that critically improved the quality of the manuscript. All authors contributed to the article and approved the submitted version.

## Conflict of Interest

The authors declare that the research was conducted in the absence of any commercial or financial relationships that could be construed as a potential conflict of interest.

## Publisher's Note

All claims expressed in this article are solely those of the authors and do not necessarily represent those of their affiliated organizations, or those of the publisher, the editors and the reviewers. Any product that may be evaluated in this article, or claim that may be made by its manufacturer, is not guaranteed or endorsed by the publisher.
