# Supplementary material for: Research on the Cognitive Diagnosis of Chinese Listening Comprehension Ability Based on the G-DINA Model
Source: Front Psychol. 2021 Sep 7;12:714568. doi: 10.3389/fpsyg.2021.714568 (PMC8452943; doi:10.3389/fpsyg.2021.714568)
Supplement: Supplementary file 4 [file Table_4.DOCX]

# APPENDIX TABLE 4

Table 4. Q-matrix-Q_4_

| Item | A1 | A2 | A3 | A4 | A5 | A6 | A7 |
| --- | --- | --- | --- | --- | --- | --- | --- |
| 1 | 1 | 0 | 0 | 1 | 0 | 0 | 0 |
| 2 | 1 | 0 | 0 | 0 | 1 | 0 | 0 |
| 3 | 1 | 0 | 0 | 0 | 0 | 0 | 0 |
| 4 | 0 | 0 | 1 | 0 | 0 | 0 | 1 |
| 5 | 1 | 0 | 1 | 1 | 0 | 0 | 0 |
| 6 | 1 | 0 | 1 | 0 | 0 | 0 | 1 |
| 7 | 1 | 1 | 1 | 0 | 1 | 0 | 0 |
| 8 | 1 | 1 | 1 | 1 | 0 | 0 | 0 |
| 9 | 0 | 0 | 1 | 0 | 0 | 1 | 1 |
| 10 | 1 | 0 | 1 | 0 | 0 | 1 | 0 |
| 11 | 0 | 0 | 0 | 1 | 0 | 0 | 1 |
| 12 | 0 | 1 | 1 | 0 | 0 | 0 | 1 |
| 13 | 0 | 1 | 1 | 0 | 0 | 0 | 1 |
| 14 | 0 | 1 | 0 | 0 | 0 | 0 | 0 |
| 15 | 0 | 1 | 1 | 0 | 1 | 0 | 1 |
| 16 | 0 | 1 | 1 | 0 | 0 | 1 | 0 |
| 17 | 0 | 0 | 1 | 0 | 0 | 0 | 0 |
| 18 | 0 | 1 | 1 | 0 | 0 | 1 | 0 |
| 19 | 0 | 1 | 0 | 0 | 0 | 0 | 0 |
| 20 | 0 | 1 | 0 | 0 | 1 | 0 | 0 |
| 21 | 1 | 0 | 1 | 0 | 1 | 1 | 0 |
| 22 | 0 | 1 | 1 | 0 | 0 | 0 | 0 |
| 23 | 0 | 1 | 0 | 1 | 0 | 1 | 1 |
| 24 | 0 | 1 | 0 | 0 | 0 | 0 | 1 |
| 25 | 0 | 0 | 1 | 1 | 0 | 0 | 1 |
| 26 | 0 | 0 | 1 | 1 | 0 | 1 | 0 |
| 27 | 0 | 0 | 0 | 1 | 0 | 0 | 0 |
| 28 | 0 | 0 | 0 | 1 | 0 | 0 | 1 |
| 29 | 0 | 1 | 0 | 1 | 0 | 1 | 0 |
| 30 | 0 | 0 | 0 | 0 | 0 | 0 | 1 |
| 31 | 0 | 1 | 0 | 0 | 1 | 0 | 1 |
| 32 | 0 | 0 | 0 | 0 | 1 | 1 | 1 |
| 33 | 0 | 0 | 0 | 0 | 1 | 0 | 0 |
| 34 | 0 | 0 | 1 | 1 | 0 | 1 | 1 |
| 35 | 0 | 1 | 0 | 0 | 1 | 0 | 0 |
| 36 | 0 | 0 | 0 | 1 | 1 | 0 | 0 |
| 37 | 0 | 0 | 0 | 1 | 0 | 1 | 0 |
| 38 | 0 | 1 | 0 | 0 | 0 | 1 | 0 |
| 39 | 1 | 0 | 0 | 1 | 0 | 1 | 0 |
| 40 | 0 | 1 | 1 | 0 | 0 | 1 | 1 |
| Total number of  measurements | 10 | 18 | 19 | 14 | 10 | 14 | 16 |
