# Supplementary material for: Research on the Cognitive Diagnosis of Chinese Listening Comprehension Ability Based on the G-DINA Model
Source: Front Psychol. 2021 Sep 7;12:714568. doi: 10.3389/fpsyg.2021.714568 (PMC8452943; doi:10.3389/fpsyg.2021.714568)
Supplement: Supplementary file 6 [file Table_6.DOCX]

# APPENDIX TABLE 6

Table 6. The likelihood ratio test

| Model | LL | Chi^2 | df | P |
| --- | --- | --- | --- | --- |
| DINA | -737665.21 | 36181.85 | 146 | <0.001 |
| DINO | -737099.68 | 35050.78 | 146 | <0.001 |
| RRUM | -723224.34 | 7300.12 | 90 | <0.001 |
