# Supplementary material for: Research on the Cognitive Diagnosis of Chinese Listening Comprehension Ability Based on the G-DINA Model
Source: Front Psychol. 2021 Sep 7;12:714568. doi: 10.3389/fpsyg.2021.714568 (PMC8452943; doi:10.3389/fpsyg.2021.714568)
Supplement: Supplementary file 7 [file Table_7.docx]

# APPENDIX TABLE 7

Table 7. Classification accuracy based on attribute classification pattern

| Attribute classification pattern | Classification accuracy | Attribute classification pattern | Classification accuracy |
| --- | --- | --- | --- |
| 0011111 | 0.0031 | 1011000 | 0.1533 |
| 1111010 | 0.0038 | 0100010 | 0.1591 |
| 0100101 | 0.0043 | 1000111 | 0.1591 |
| 0011011 | 0.0113 | 1001100 | 0.1593 |
| 0011000 | 0.0177 | 0100100 | 0.1744 |
| 0000010 | 0.0224 | 0001010 | 0.1813 |
| 0001011 | 0.0252 | 0011110 | 0.2259 |
| 1011110 | 0.0277 | 1110000 | 0.2297 |
| 1011011 | 0.0473 | 1011001 | 0.2321 |
| 0100000 | 0.0491 | 0000101 | 0.2430 |
| 1011100 | 0.0610 | 0111111 | 0.2800 |
| 0100001 | 0.0626 | 1001001 | 0.3026 |
| 0111011 | 0.0673 | 1001000 | 0.3489 |
| 0110001 | 0.0803 | 0001101 | 0.3593 |
| 1011101 | 0.0940 | 1101111 | 0.3780 |
| 0001110 | 0.0983 | 0001001 | 0.4746 |
| 0111110 | 0.1119 | 0110110 | 0.5094 |
| 1010000 | 0.1168 | 1110010 | 0.5360 |
| 1100000 | 0.1244 | 1110101 | 0.5939 |
| 0011010 | 0.1254 | 0011100 | 0.7786 |
| 0000001 | 0.1332 | 0100011 | 0.7898 |
| 1110001 | 0.1338 | 0000000 | 0.9177 |
| 1001110 | 0.1488 | 1111111 | 0.9259 |
