# Supplementary material for: Research on the Cognitive Diagnosis of Chinese Listening Comprehension Ability Based on the G-DINA Model
Source: Front Psychol. 2021 Sep 7;12:714568. doi: 10.3389/fpsyg.2021.714568 (PMC8452943; doi:10.3389/fpsyg.2021.714568)
Supplement: Supplementary file 8 [file Table_8.DOCX]

# APPENDIX TABLE 8

Table 8. Tetrachoric correlation among the attributes

| attributes | A1 | A2 | A3 | A4 | A5 | A6 | A7 |
| --- | --- | --- | --- | --- | --- | --- | --- |
| A1 | 1 | 0.693** | 0.637** | 0.258** | 0.647** | 0.383** | 0.704** |
| A2 |  | 1 | -0.105** | -0.347** | -0.097** | 0.769** | 0.997** |
| A3 |  |  | 1 | 0.797** | 0.996** | -0.213** | -0.091** |
| A4 |  |  |  | 1 | 0.776** | -0.029** | -0.337** |
| A5 |  |  |  |  | 1 | -0.225** | -0.077** |
| A6 |  |  |  |  |  | 1 | 0.763** |
| A7 |  |  |  |  |  |  | 1 |
